# Adaptive Vaccination Strategies to Mitigate Pandemic Influenza: Mexico as a Case Study

**DOI:** 10.1371/journal.pone.0008164

**Published:** 2009-12-03

**Authors:** Gerardo Chowell, Cécile Viboud, Xiaohong Wang, Stefano M. Bertozzi, Mark A. Miller

**Affiliations:** 1 Mathematical, Computational & Modeling Sciences Center, School of Human Evolution and Social Change, Arizona State University, Tempe, Arizona, United States of America; 2 Fogarty International Center, National Institutes of Health, Bethesda, Maryland, United States of America; 3 National Institute of Public Health, Center for Evaluation Research and Surveys, Cuernavaca, Mexico; 4 University of California, Berkeley, California, United States of America; University of Nebraska, United States of America

## Abstract

**Background:**

We explore vaccination strategies against pandemic influenza in Mexico using an age-structured transmission model calibrated against local epidemiological data from the Spring 2009 A(H1N1) pandemic.

**Methods and Findings:**

In the context of limited vaccine supplies, we evaluate age-targeted allocation strategies that either prioritize youngest children and persons over 65 years of age, as for seasonal influenza, or adaptively prioritize age groups based on the age patterns of hospitalization and death monitored in real-time during the early stages of the pandemic. Overall the adaptive vaccination strategy outperformed the seasonal influenza vaccination allocation strategy for a wide range of disease and vaccine coverage parameters.

**Conclusions:**

This modeling approach could inform policies for Mexico and other countries with similar demographic features and vaccine resources issues, with regard to the mitigation of the S-OIV pandemic. We also discuss logistical issues associated with the implementation of adaptive vaccination strategies in the context of past and future influenza pandemics.

## Introduction

Although countries have developed influenza pandemic preparedness plans, uncertainties remain in terms of the virulence and transmissibility of pandemic strains as well as population immunity profiles. In particular, there has been heterogeneity in the past three influenza pandemics of the 20th Century [1], [2] with regard to transmissibility, ranging from an average of 1.5 to 5.4 secondary cases per primary case in the community; [3], [4], [5], [6], [7]; case fatality rate, range, 0.1%–4% [3], [8] ; and age-specific mortality rates [3], [9]. While differences in transmissibility and case fatality rate remain poorly understood, mortality age patterns could be explained in part by the history of previously circulating influenza viruses, with early-life exposure to related viruses reducing risk for severe pandemic outcomes [10], [11]. Moreover, recent studies have evidenced important geographical variations in pandemic morbidity and mortality burden [2], [7], [12], [13], [14], [15], as well as variations in severity of successive pandemic waves. Pandemic preparedness plans have not adequately incorporated such uncertainties, which are difficult to resolve prior to pandemic onset but can be deduced once a novel pandemic virus is identified.

Given the variety of possible pandemic scenarios, specific information on virus sub-type and age patterns of incidence and mortality during the early phase of a pandemic could help prioritize allocation of limited resources and optimize reductions in disease burden. Containment [16], [17], [18], [19] and control strategies for influenza pandemics have been explored by simulations and applied to several countries or regions, including Southeast Asia [16], [18], US [20], [21], [22], [23], UK [23], and Netherlands [22], [23], [24]. None of these simulations featured adaptive intervention strategies that integrate epidemiological data collected in real time during the first weeks of the outbreak. The recent emergence of a novel swine-origin influenza A/H1N1 virus (S-OIV) in Mexico [25] and rapid global spread [26] provides an opportunity for modeling in a real-time pandemic situation and may provide guidance for public health officials in many countries.

A(H1N1) S-OIV pandemic influenza virus continues to spread throughout the Northern and Southern hemispheres. While there are plans to formulate a vaccine against the new A(H1N1) strain, current licensed manufacturing processes are insufficient to protect the majority of the six plus billion people who may be potentially exposed during the first pandemic wave. Through a review of the epidemiology thus far and principles of past pandemics, vaccine efficacy and transmissibility factors within and between age groups, we provide an optimization strategy to minimize severe morbidity and mortality burden from this virus. Specifically, we evaluate the effectiveness of various age-targeted vaccination strategies against pandemic influenza when vaccine supplies are limited. We propose novel ‘adaptive’ real-time vaccination strategies that may guide vaccine allocation based on the age patterns of morbidity and mortality of an on-going outbreak, and compare their effectiveness with that of strategies targeted towards traditional influenza high risk age groups, i.e., young children and seniors. We calibrate our models against local demographic and epidemiological data from the 2009 outbreak of S-OIV in Mexico in an emerging scenario and explore a moderate pandemic scenario illustrating the epidemiology of the 1957- or 1968 pandemics and a severe pandemic scenario illustrating the unusual concentration of hospitalizations and deaths in young adults observed during the 1918-pandemic.

## Materials and Methods

To compare the effectiveness of various vaccination strategies against pandemic influenza in Mexico, we used an age-structured influenza transmission model that accounts for age-specific risk of illness, hospitalization, and death and simulated a variety of epidemiological and vaccination scenarios encompassing the diversity of observed disease patterns from previous pandemics. Incidence rates of clinical cases, hospitalizations and deaths in the absence of vaccination were estimated based on the outbreak of S-OIV in Mexico. In this simulation approach, we assume limited vaccine supplies and age variation in vaccine efficacy. Below we describe the structure of the transmission model, discuss the pandemic scenarios considered, and detail the different vaccination strategies evaluated.

### Transmission Model

**Figure 1:**
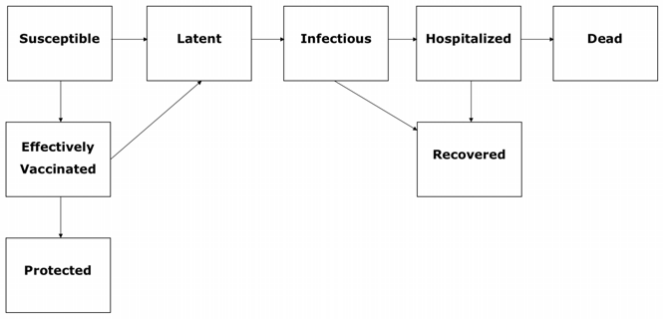
Flow chart of the stage progression of the individuals among the different epidemiological classes.

Figure 1 provides a schematic of the transmission model that includes 6 age groups (0–5 y, 6–12 y, 13–19 y, 20–39 y, 40–59 y, > = 60 y). We integrate the age structure of the Mexican population to the influenza transmission model, based on data from the 2000 Census (Figure S1). Further, each age group (indexed by *i*) is classified into 9 epidemiological states given by: susceptible (S_i_), effectively vaccinated but not yet protected (V_i_), ineffectively vaccinated (U_i_), protected by vaccination (P_i_), latent (E_i_), symptomatic and infectious in the community (I_i_), hospitalized (H_i_), recovered (R_i_), and dead (D_i_). Susceptible individuals in age group *i* are exposed to the influenza virus at the force of infection 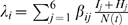 where 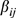
is the transmission rate between age groups *i* and *j* and the total population size is given by
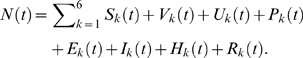



The transmission rates 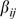 are given by qc_ij_ where q is the transmission probability per contact (fraction of contacts that leads to infection), which is assumed to be constant across age groups as in previous studies [24], and c_ij_ are the age-specific contact rates which are modeled based on a study describing self-reported age-specific contact rates for the spread of respiratory infections [27] (Table S1). Although information on contact rates is limited [28], transmission models calibrated with frequency dependent contact rates derived from social survey have been shown to provide better approximations to attack rates of the 1957 influenza pandemic than other mixing assumptions[27]. The contact rate matrix is highly assortative with higher mixing rates within each age group than between age groups and follows a similar pattern to that of several European countries [29]. Contact rates among 6–12 year olds are the highest and rates among seniors (> = 60 y) are the lowest. Latent individuals E_i_ progress to the infectious class I_i_ at the rate k (1/k is the mean latent period). Infectious individuals are hospitalized at the age-specific mean rates α_i_ and recover at the mean rate γ_1_. Hospitalized individuals either recover at the constant rate γ_2_ or die from influenza at the age-specific rate δ_i_. While the age-specific hospitalization rates are adjusted using estimates of the probability of hospitalization given clinical illness by age group, the recovery rate γ_2_ is assumed to be constant across age groups for simplicity. Recovered individuals are assumed to remain protected for the duration of the epidemic. Infected individuals die with an age-specific mortality rates as described below. Vaccination is administered to susceptible individuals t^*^ days after the epidemic onset with a vaccination rate ν(t). That is, ν(t) = 0 whenever t < t^*^. Age-specific vaccine efficacy is denoted by 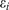. Successfully vaccinated individuals (V_i_) progress to the protected class (P_i_) at the rate η (mean of 10 days) while ineffectively vaccinated individuals (U_i_) remain susceptible to infection. Vaccinated but not yet protected individuals (V_i_) may still be infected with influenza at the age-dependent force of infection λ_i_ as described above. The population is assumed to be completely susceptible at the beginning of the epidemic. The system of differential equations that describes our influenza transmission model is given by
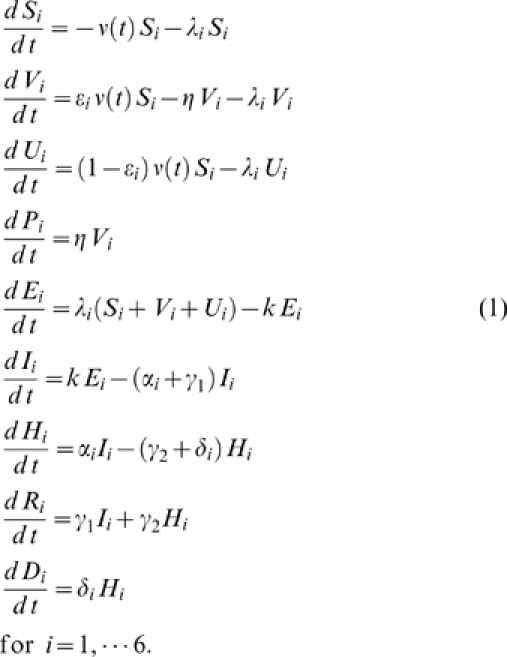



The hospitalization and mortality rates are given by α_i_ = (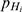/(1 − 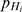))γ_1_ and δ_i_ = (*CFP*_i_/(1−*CFP*_i_)) γ_2_, respectively, where 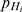 and *CFP*_i_ denote the probability of hospitalization given clinical illness and the probability of death following hospitalization for age group *i* (Figures S2, S3). Numerous simulations of the model were conducted by solving the system of ordinary differential equations using Matlab (The Mathworks, Inc) with initially 5 infectious teenagers (13–19) following the different pandemic scenarios.

### Model Calibration and Reproduction Number Estimates

The basic reproduction number R_0_, the average number of secondary cases generated by a primary infectious case during the initial epidemic period [30], [31], quantifies the transmissibility of a pathogen in naïve populations, while the reproduction number, R, estimates a similar quantity in partially-naïve populations. These estimates can help determine the intensity of interventions necessary for epidemic control. If the average number of secondary infections is reduced then transmission slows down (transmission is interrupted if R<1), so that there is less pressure on health care systems and potentially increased time to prepare additional vaccines. We derive an expression of the reproduction number from our mathematical model using standard methods [31] and calibrate our model against published estimates of the basic reproduction number for this influenza pandemic in Mexico by adjusting the probability of transmission given contact (q) (File S1).

### Adaptive and Seasonal Vaccination Strategies

Vaccination strategies considered in this study are implemented after the onset of an influenza pandemic outbreak. We considered 2 strategies, depending on the age targets for vaccination: 1. a ‘seasonal-like influenza vaccination strategy’ targeting the same high-risk groups as for seasonal vaccination, young children (0–5 y) and seniors (> = 60 y) 2. an ‘adaptive’ strategy, where vaccine is allocated on the basis of age-specific hospitalization and mortality rates reported in real-time during the early pandemic phase. Vaccine doses are allocated to age groups proportionally to their corresponding hospitalization or mortality rates. Benefits are potentially optimized if early reports of age-specific hospitalization and death are indicative of those at risk throughout all phases of a pandemic.

We assume that timing of vaccine delivery follows an exponential distribution with an average of 5 days after the vaccination campaign is initiated. Vaccine efficacy is assumed to be 77.5% (range between 75% and 80%) for individuals <65 y and 35% (range between 17% and 53%) for seniors over 65 years, based on reviews of influenza vaccine immunogenicity [32], [33], [34]. Vaccinated individuals are assumed to develop protection 10 or 30 days after immunization on average depending on the requirements of one or two doses of a novel vaccine. Because vaccine is likely in limited supply during the earliest phase of a pandemic, the vaccination coverage with the full course of 1–2 doses is assumed to be relatively low (5%–20%). Our upper bound for vaccination coverage is consistent with the immunization of about one fifth of the US population in 1976 against a swine influenza virus [35]. We assume that the same vaccination strategy will be applied throughout the outbreak or until vaccines resources are depleted.

### Pandemics Scenarios

Model parameters describing the epidemiology of pandemic influenza are given in Table 1. We considered 3 pandemic scenarios, representing the epidemiology of past and current pandemics, as explained below.

**Table 1:** Parameter definitions and mean baseline values of influenza epidemiology used in our transmission model.

Parameter	Definition	Baseline values	Source
k	Rate of progression from latent to infectious state (1/day)	1/1.9	[19]
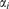	Diagnostic rate for age group *i* (1/day)		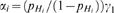
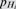	Fraction of clinical cases that are hospitalized for age group *i*	Figure 2, Figure S2	[36], [37], [38]
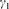	Recovery rate for infectious class (1/day)	1/1.5 (1/4–1)	[4]
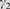	Recovery rate for hospitalized class (1/day)	1/1.5 (1/4–1)	[4]
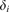	Influenza mortality rate for age group *i* (1/day)		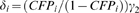
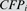	Case fatality proportion	Figure 2, Figure S3	[36], [38]

#### a) 2009 H1N1 pandemic scenario

**Figure 2:**
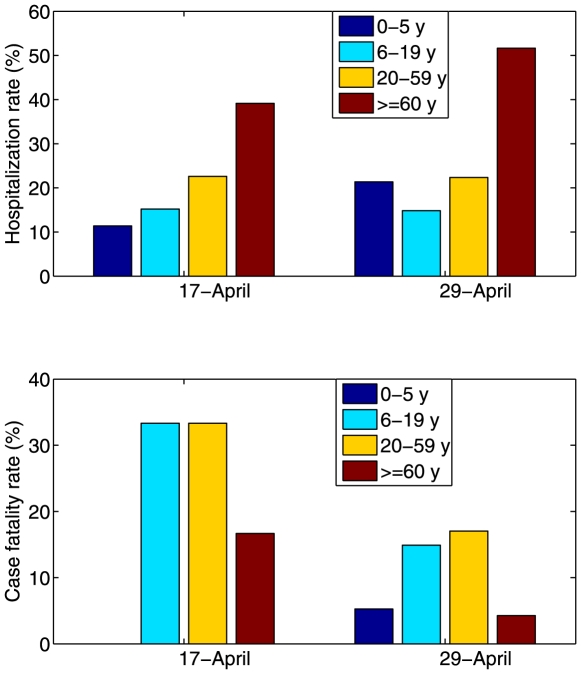
Age-specific hospitalization and case fatality rates given hospitalization estimated from cumulative morbidity and mortality data from the National Surveillance System stratified by four broad age groups at two time points into the S-OIV outbreak in Mexico. The Ministry of Health issued an epidemiologic alert on April 16 and 17 and selective reporting of severe pneumonia cases ceased on April 29. Case fatality rate is estimated as the proportion of deaths among hospitalized pneumonia cases.

To model an epidemiological pandemic scenario reminiscent of the H1N1 outbreak in Mexico in Spring 2009, we used age-specific epidemiological data reported to the Mexican National Epidemiological Surveillance System during this outbreak [36]. Age-specific hospitalization and case fatality rates for hospitalized cases (age groups 0–4 y, 5–59 y, > = 60 y) were estimated from cumulative morbidity and mortality data reported on two epidemiologically-relevant dates of the outbreak: (i) on April 17, 25 days into the outbreaks (March 24 -April 17, 2009), when the Ministry of Health requested that medical institutions intensify notification and (ii) on Apr 29, 2009, 37 days into the outbreak, when selective reporting of pneumonia requiring hospitalization ceased [25] (Figure 2). We varied the mean reproduction number within its estimated range 1.4–1.8 for this outbreak [26] and vaccination coverage levels of 5–20%.

#### b) Historical pandemic scenarios

We considered two more scenarios illustrating the age patterns of hospitalizations and deaths during a pandemic: (i) a moderate pandemic scenario illustrating the epidemiology of the 1957- or 1968 pandemics, with increased severe outcomes in young children and seniors [37] and (ii) a severe pandemic scenario illustrating the unusual concentration of hospitalizations and deaths in young adults observed during the 1918-pandemic [38] (Figure S2).

For the 1957- and 1968-like moderate pandemic scenarios, we estimated age- specific case fatality rates for hospitalized influenza cases by combining hospitalization (Figure S4) and mortality data from the city of Guadalajara, Mexico during the period 2000–2005 (Ministry of Health, State of Jalisco, Mexico, Figure S5). For the 1918-like severe pandemic scenario, we used historical data [38] to estimate case fatality rates, which were highest among young adults (20–39 year-old, Figure S3). The case fatality rate is likely overestimated for this scenario due to secular improvements in health care since the 1918 pandemic; however we consider this simulation a worst-case scenario.

## Results

### 2009 A/H1N1 Pandemic Scenario, Based on the Epidemiology of Influenza in Mexico, Spring 2009

**Figure 3:**
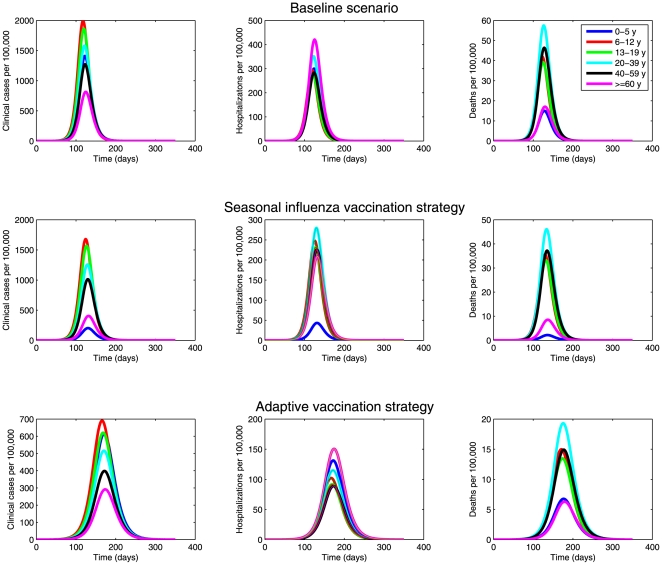
Age-specific incidence curves of clinical cases, hospitalizations and deaths in the context of the 2009 S-OIV outbreak in Mexico. We considered a baseline situation with R_0_ = 1.6 where no vaccine is used (top panel), a seasonal vaccination strategy (middle panel) where priority groups for vaccination are young children and seniors, as for seasonal influenza, and an adaptive vaccination strategy (bottom panel) where vaccine is allocated according to data on age-specific rates of mortality. The vaccination coverage is 20% and vaccination is initiated 25 days after the epidemic onset when an epidemiologic alert was issued in Mexico.

First, we explored a baseline pandemic scenario reminiscent of the recent Mexican experience with novel S-OIV in the spring of 2009, in the absence of any intervention. Assuming R_0_ = 1.6, the peak was reached at about day 120 after the pandemic onset with a total outbreak duration of about 5 months. Hospitalization rates varied from about 10% for the 13–19 year-olds to 17% for the > = 60 year-olds as shown in Figure 3 (top panel). Mortality rates were estimated at about 2.3-, 1.9- and 0.7-percent for year age groups 20–39, 13–19, and > = 60 y, respectively.

**Figure 4:**
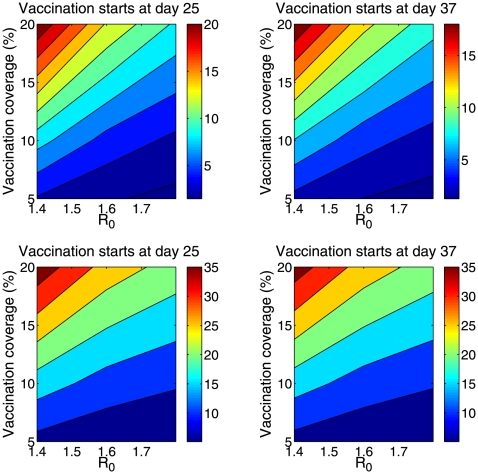
Comparison of concurrent vaccination strategies for novel S-OIV pandemic influenza scenarios. Strategies include adaptive vaccination targeted at high-risk groups identified from real-time hospitalization and death data, and seasonal vaccination strategy targeted at young children and seniors. Plots illustrate the additional reduction (%, see color bar) in hospitalizations (top) and deaths (bottom) averted by the adaptive vaccination allocation strategy, compared to the seasonal influenza strategy, as a function of R (using plausible ranges, R = 1.4–1.8) and vaccination coverage (5–20%). In the left panels, vaccination is initiated 25 days into the outbreak, on April-17-2009, when the epidemiologic alert was issued. In the right panels, vaccination is initiated 37 days into the outbreak, on Apr-29-2009, when selective reporting of severe pneumonia cases ceased. The adaptive strategy allocates vaccine given the age-specific patterns of hospitalization and mortality available on those dates (Figure 2).

To define priority groups for the adaptive strategy, we relied on early estimates of age-specific rates of hospitalization and death, given epidemiological data reported 25 and 37 days into the outbreak (Apr 17 and Apr 29; Fig. 2). Assuming R_0_ = 1.6 (Table 2), the adaptive strategy yielded reductions of 37% and 42% in the overall number of hospitalizations and deaths (Figure 3 bottom panel), respectively, if vaccination started on day 25 of the outbreak and reached 20% of the population. The benefits of the adaptive strategy were slightly lower if vaccination started on day 37 of the outbreak and reached 20% of the population. The adaptive vaccination strategy provided up to 35% additional reduction in the number of influenza-related deaths and 22% reduction in hospitalization as compared to the seasonal influenza strategy (Figures 3), for transmissibility levels of 1.4–1.8 and vaccination coverage ranging between 5 and 20% (Figure 4). Given the age patterns of hospitalizations and deaths in the S-OIV Mexican pandemic scenario, the age group prioritized by the adaptive strategy was young and middle-aged adults aged 20–59 y.

**Table 2:** Comparison of predicted rates (per 100,000 people) of hospitalizations and death in the context of the 2009 S-OIV outbreak in Mexico, for various concurrent vaccination strategies.

	Baseline (no interventions)	Seasonal vaccination strategy		Adaptive vaccination strategy	
		Vaccination coverage 5%	Vaccination coverage 20%	Vaccination coverage 5%	Vaccination coverage 20%
**Hospitalizations**	12202	11357 (7%)	9395 (23%)	11085 (9%)	7714 (37%)
**Deaths**	1665	1593 (4%)	1419 (15%)	1490 (11%)	954 (43%)

We considered a baseline situation where no vaccine is used; a seasonal vaccination strategy where priority groups for vaccination are young children and seniors, as for seasonal influenza; and an adaptive vaccination strategy where vaccine is allocated according to data on age-specific rates of mortality and hospitalization with a baseline R_0_ = 1.6 [26] and vaccination coverage is 5 or 20%. Vaccination is initiated 25 days after the epidemic onset when an epidemiologic alert was issued in Mexico.

### Historical Pandemic Scenarios

For the severe pandemic scenario illustrating the epidemiology of the 1918 pandemic with R_0_ = 1.8 (clinical attack rate across the population of 39%), the peak was reached at about day 85 after the pandemic onset with a total outbreak duration of about 4 months. Hospitalization rates varied from about 2,750 per 100,000 individuals for the 40–59 year-olds to 4,250 per 100,000 for the 0–5 year-olds as shown in Figure S6 (top panel). Mortality rates were estimated at about 500, 420 and 290 deaths per 100,000 for year age groups 20–39, 0–5, and > = 60 y, respectively.

The moderate pandemic scenario illustrating the epidemiology of the 1957- and 1968-pandemics produced similar peak timings and outbreak duration as the 1918-like scenario, for a given transmissibility level. As expected, the moderate scenario resulted in lower hospitalization rates, varying from about 75 per 100,000 individuals for the 6–12 y and 13-19 year-olds to 3,150 per 100,000 for the > = 60 year-olds when R_0_ = 1.8 (Figure S7 top panel). Mortality rates were below 9 per 100,000 for all age groups except for > = 60 year olds with about 475 deaths per 100,000 individuals.

For illustration purposes, we compare the effectiveness of the “seasonal” influenza vaccination strategy targeting young children and seniors to mitigate disease in severe and moderate pandemic scenarios (Figures S6 and S7). For the moderate 1957 or 1968-like pandemic scenario, the “seasonal” vaccination strategy yielded reductions of 32% and 37% in the overall number of hospitalizations and deaths, respectively, if vaccination started on day 10 of the outbreak and reached 20% of the population. The corresponding reductions for a severe 1918-like pandemic scenario were 20% and 24%, respectively.

Next, we considered the effectiveness of adaptive strategies integrating real-time epidemiological data. For the moderate pandemic scenario, the adaptive strategy yielded reductions of 49% and 43% in the overall number of hospitalizations and deaths, respectively, if vaccination started on day 10 of the outbreak and reached 20% of the population. Overall, the adaptive vaccination strategy outperformed the seasonal strategy in terms of reducing hospitalizations and deaths for all values of R and the entire range of vaccination coverage (5–20%). The adaptive vaccination strategy gave 2–19% additional reduction in hospitalization and 1–20% additional reduction in deaths, compared to the seasonal influenza vaccination strategy, when vaccination was initiated 10 days after pandemic onset. The superiority of the adaptive strategy held when the vaccination campaign was initiated 30 days after the epidemic onset (Figure S8). Overall, the adaptive vaccination strategy substantially outperformed the seasonal influenza vaccination strategy if vaccination coverage was relatively high (>10%) and transmissibility remained relatively low (R<3); additional benefits quickly decreased when R was greater than 3 or vaccination coverage was less than 10% (Figure S8). In addition, the added benefits of the adaptive strategy increased when the vaccination campaign started early. For the moderate pandemic scenario illustrating the epidemiology of the 1957- and 1968-pandemics, the age groups prioritized by the adaptive strategy were adults aged 60 and over.

For a severe 1918-like pandemic scenario, the improvements of the adaptive vaccination strategy over the seasonal influenza vaccination strategy were significantly greater than those obtained for the moderate pandemic scenario. For the severe pandemic scenario, the adaptive strategy yielded reductions of 36% and 37% in the overall number of hospitalizations and deaths, respectively, if vaccination started on day 10 of the outbreak and reached 20% of the population. The additional benefits of the adaptive vaccination strategy ranged from 1–55% added reduction in hospitalization and 1–29% reduction in deaths, compared with the seasonal influenza vaccination strategy. The largest added benefits of the adaptive strategy were found at higher vaccination coverage, lower R values, and earlier start of vaccination campaigns, in line with results for the moderate pandemic scenario. For the severe pandemic scenario illustrating the epidemiology of the 1918 pandemic, the age group prioritized by the adaptive strategy was adults aged 20–39 y followed by 40–59 year-olds.

## Discussion

With today's technologies, little to no vaccines would be immediately available for most of the world at the time of emergence of a novel pandemic virus to contain a potential “herald” wave, as was observed during the summer of 1918 in the US and Europe [4], [7], [39]. A similar situation occurred with the first wave of the S-OIV pandemic in Mexico and elsewhere in spring 2009. Previous models [16], [17], [18] predicted that containment of pandemic influenza could not succeed unless multiple medical and non-medical interventions were layered and applied early, a low to zero probability scenario given the rapidity of events in influenza transmission and global spread. Given the time line of vaccine production and delivery for pandemic viruses, a realistic use of vaccination would be its concurrent delivery during an ongoing pandemic, in particular during a second or third wave.

In this study, an age-structured model of influenza transmission, hospitalization and death, was used to explore the effectiveness of various age-targeted vaccination strategies in pandemic scenarios reminiscent of the epidemiology of the novel S-OIV A/H1N1 outbreak in Mexico in the spring of 2009 and past pandemics. The model integrates age variation in vaccine efficacy and probability of severe disease outcomes, as well as direct and indirect benefits of vaccination. The adaptive vaccination strategy relying on data on hospitalization and deaths reported as early as 25 days into the outbreak was the most effective to reduce deaths and hospitalizations when vaccine resources were scarce and substantially outperformed the seasonal influenza allocation strategy for a range of parameter values. In particular, the adaptive strategy would provide additional reductions of up to 22% and 35% in hospitalizations and deaths compared to the ‘seasonal’ vaccination strategy targeting traditional high-risk groups, at 20% vaccine coverage. This assumes that the population between 20 and 59 years would be preferentially targeted, in contrast to seniors and young children who are traditionally prioritized. Although a variety of alternative vaccine allocation schemes are possible, here we focused on the seasonal strategy as a reference baseline strategy, since it is the most widely used to control seasonal influenza globally.

Similar benefits were obtained for other pandemic scenarios illustrating the epidemiology of the 1918, 1957 and 1968 pandemics, and a wider range of R_0_ values. The adaptive strategy produced 2–20% additional reductions in deaths and hospitalizations when applied to the 1918 pandemic scenario, relative to the seasonal allocation strategy, for the range of parameters considered. It is interesting that both the 1918 and 2009 pandemics have particularly young age distribution of severe cases: while the 1918 pandemic caused mortality mostly in persons below the age of 45 [9], [39], the current 2009 H1N1 pandemic affects mostly those under 55 [25]. Because of the pronounced shift in pandemic deaths towards younger age groups in those pandemics, the benefits of the adaptive strategy are enhanced as compared to the seasonal strategy.

Direct and indirect effects of vaccination are incorporated in this study, including differential rates of transmission within and between age-groups and age-variation in vaccine efficacy. While school-age children amplify influenza transmission locally, adults are likely responsible for inter-regional spread [40], including in the current H1N1 pandemic [41]. We note that interruption of transmission was not achieved in any of the vaccination scenarios considered here, including those targeting high-transmitter groups. This is likely because we explored low vaccination coverage, never exceeding 20%, and vaccination was assumed to start well into the outbreak in most scenarios.

An adaptive vaccination strategy requires rapid ascertainment of cases, hospitalizations and/or deaths, to help identify high risk age groups for prioritization of vaccine and other pharmaceutical interventions, including antivirals and antibiotics [42], [43]. In the 2009 swine flu outbreak, the optimal strategy could be identified with confidence as early as day 25 of the Mexican outbreak, given knowledge on the age pattern of severe cases and local availability of real-time data. Had a vaccine been available in quantities sufficient to cover 20% of the population, this would have given enough time to initiate a vaccination campaign and avert an additional 22% of hospitalizations and 35% of deaths compared to a seasonal vaccination strategy if vaccination started 25 days after the epidemic onset. Alternatively, if local epidemiological data are not available in real time, data from other countries experiencing earlier or simultaneous outbreaks could be used to calibrate the adaptive strategy. Such a strategy might be particularly useful in the case of returning outbreaks of S-OIV in the fall, in Mexico and elsewhere.

Virological subtyping of a novel pandemic virus can provide an early clue to target vaccination efforts. Each of the previous pandemics had unique age-mortality patterns [9] that could be explained by previous exposure during childhood of a subset of the population to the novel circulating viral sub-type [1], [32], [10]. While the elderly are normally at most risk for severe outcomes during seasonal influenza, warranting the targeting of vaccination for direct protection to that group, they may have residual protection during pandemics. By contrast, younger groups generally respond better to vaccine [34], [33] and provide a greater reduction of transmission. Given residual protection in seniors in early pandemic waves, younger age groups become a clear priority group for pandemic vaccine allocation. In the current 2009 pandemic, assuming an annual attack rate of approximately 10–20% for inter-pandemic influenza, those who were born between 1919 and around 1957 would have been first exposed to H1N1 during their childhood and may enjoy protection against S-OIV infection and death, as observed in the early wave of S-OIV in Mexico [25].

Several studies have assessed the effects of potential vaccination strategies against pandemic influenza [24], [44], [45], [46], [47], [48], [49] in terms of reducing morbidity and mortality based on priority age groups, transmissibility, timing of vaccination efforts [24], and number of years of life lost [45]. A recent study [24] has evaluated the influenza vaccine allocation problem considering a vaccination coverage of 35% at the pandemic onset or near the pandemic peak when the population is stratified by age and low and high risks. Results suggest that vaccine should be allocated to individuals with high-risk complications whenever the vaccine becomes available late in the pandemic (close to the peak) while targeting high transmitter groups (children) is more effective when the vaccine is available close to the start of the pandemic. Most studies of influenza vaccination strategies to date have assumed a given epidemiological profile based on past influenza epidemics and pandemics but have not necessarily considered novel profiles that could arise in future pandemics. Given high levels of uncertainty as to the epidemiology of the next outbreak of S-OIV or other novel influenza virus, unfortunately, no single strategy can fit all scenarios. Our adaptive strategy is flexible enough to accommodate a range of possible scenarios illustrating our experience with past pandemics, and potentially new ones.

We note that other interventions strategies have been proposed to mitigate the burden of pandemic influenza. Social distancing and facemasks have been suggested as mitigation strategies, but their efficacy against pandemics remains debated. Strategies involving antiviral treatments are helpful to mitigate disease burden, but resources are limited and effectiveness assumes speedy delivery and susceptibility of circulating viruses. Any of these interventions could be used in combination with the adaptive vaccine allocation strategy proposed here.

Mexico began vaccinating against seasonal influenza in 2004, and annual campaigns target children 6 to 23 months old, adults over 65 years, and those with chronic conditions [50]. In the past, Mexico has relied on other countries for influenza vaccine production, which in the setting of a pandemic is likely to be available in limited supplies. Although a preparedness and response plan against pandemic influenza for Mexico had been drafted with the objective of optimizing resources and conducting a timely response [51], [52], it lacks guidance on how to define priority groups in the scenario of a limited vaccine supply. Our study shows that even limited vaccine supplies, if used optimally, can have an impact on mitigating disease burden in a middle-income country like Mexico.

There are many limitations to policy models with respect to choice of parameter estimates and the incorporation of bio-medical, environmental, operational, political, economic features. No one model can claim to incorporate all assumptions and features given the limited data on the epidemiology of novel pandemic viruses and paucity of data on contact rates, especially in Mexico. This model illustrates a prioritization scheme based on age-groups but does not further discriminate other sub-groups such as those persons with other medical conditions including pregnancy. Models do not necessarily provide answers but help articulate the questions, assumptions and numerous uncertainties in rapidly evolving circumstances as a tool to formulate rational policy based on the best available evidence. Pandemics evolve rapidly relative to capabilities to enact policies; therefore, pre-formulated adaptive strategies can readily take into account new data. Knowledge of the specific sub-type circulating and real-time information on age-specific rates of severe outcomes are crucial to help policy makers infer who may be at most risk, and tailor intervention strategies accordingly. These adaptive pandemic strategies could be readily adopted by other countries.

## Supporting Information

Figure S1 Age distribution of Mexico's population according to the 2000 census
(0.01 MB EPS)

Figure S2 The age-specific probabilities of hospitalization given clinical illness under the moderate pandemic scenario based on ref. [37] except for estimates in the elderly and the 1918 influenza pandemic profile which are based on US data [38].
(0.01 MB EPS)

Figure S3 The age-specific case fatality rates given hospitalization for the typical influenza profile using hospitalization and mortality data from the city of Guadalajara, Mexico during the period 2000–2005 (Ministry of Health, State of Jalisco, Mexico) and the 1918 influenza pandemic profile.
(0.01 MB EPS)

Figure S4 Weekly number of pneumonia related hospitalizations in the city of Guadalajara, Mexico during the period 2000–2007 for three age groups (Ministry of Health, State of Jalisco, Mexico).
(0.02 MB EPS)

Figure S5 Case fatality rate (CFP) as measured by the ratio of age-specific hospitalizations and deaths due to pneumonia and influenza (P\&I) in the city of Guadalajara, Mexico for years 2000–2005 (Ministry of Health, State of Jalisco, Mexico).
(0.02 MB EPS)

Figure S6 Age-specific incidence curves of clinical cases, hospitalizations and deaths for a baseline scenario with the 1918-like pandemic profile without vaccination with R_0 = 1.8 (top panel) and the impact of a seasonal influenza vaccination strategy with R_0 = 1.8, start of vaccination at day 10 of epidemic onset, average per-capita time to vaccination of 5 days and a vaccination coverage of 20% (bottom panel).
(0.06 MB EPS)

Figure S7 Age-specific incidence curves of clinical cases, hospitalizations and deaths for a baseline scenario under the moderate pandemic scenario of hospitalization and mortality without vaccination with R_0 = 1.8 (top panel) and the impact of a seasonal influenza vaccination strategy with R_0 = 1.8, start of vaccination at day 10 of epidemic onset, average per-capita time to vaccination of 5 days and a vaccination coverage of 20% (bottom panel).
(0.06 MB EPS)

Figure S8 Comparison of concurrent vaccination strategies for 1918-like and typical pandemic influenza scenarios. Strategies include adaptive vaccination targeted at high-risk groups identified from hospitalization and death data, and seasonal vaccination strategy targeted at young children and seniors. Reduction (%) in hospitalizations (top) and deaths (bottom) averted by the adaptive vaccination allocation strategy compared to the seasonal influenza strategy as a function of R and vaccination coverage (%) under the moderate pandemic scenario (left) and the characteristic 1918 influenza pandemic profile (right) of hospitalization and mortality when vaccination starts 30 days after the epidemic onset.
(0.03 MB EPS)

Table S1 Normalized age-specific contact rates c_(i,j) per week as estimated from self-reported data for a typical week, after correction for reciprocity, Utrecht, the Netherlands, 1986 [27].
(0.02 MB PDF)

File S1 Derivation of the basic reproduction number
(0.04 MB PDF)
