# Supplementary material for: Adaptive Vaccination Strategies to Mitigate Pandemic Influenza: Mexico as a Case Study
Source: PLoS One. 2009 Dec 3;4(12):e8164. doi: 10.1371/journal.pone.0008164 (PMC2781783; doi:10.1371/journal.pone.0008164)
Supplement: Table S1 — Normalized age-specific contact rates c_(i,j) per week as estimated from self-reported data for a typical week, after correction for reciprocity, Utrecht, the Netherlands, 1986 [27]. (0.02 MB PDF) [file pone.0008164.s009.pdf]

Table S1: Normalized age-specific contact rates $c_{i,j}$ per week as estimated from self-reported data for a typical week, after correction for reciprocity, Utrecht, the Netherlands, 1986 [27].

Age class (years) of contacts	Age class (years) of participant					
	0-5	6-12	13-19	20-39	40-59	$\geq$60
0-5	169.14	31.47	17.76	34.50	15.83	11.47
6-12	31.47	274.51	32.31	34.86	20.61	11.50
13-19	17.76	32.31	224.25	50.75	37.52	14.96
20-39	34.50	34.86	50.75	75.66	49.45	25.08
40-59	15.83	20.61	37.52	49.45	61.26	32.99
$\geq$ 60	11.47	11.50	14.96	25.08	32.99	54.23
