# Supplementary material for: Adaptive Vaccination Strategies to Mitigate Pandemic Influenza: Mexico as a Case Study
Source: PLoS One. 2009 Dec 3;4(12):e8164. doi: 10.1371/journal.pone.0008164 (PMC2781783; doi:10.1371/journal.pone.0008164)
Supplement: File S1 — Derivation of the basic reproduction number (0.04 MB PDF) [file pone.0008164.s010.pdf]

Supplementary information

G. Chowell[1,2], C. Viboud[2], X. Wang[1], S.M. Bertozzi[3,4], M. A. Miller[2]

[1] Mathematical, Computational & Modeling Sciences Center,

School of Human Evolution and Social Change, Arizona State University

Tempe, AZ 85282, USA

[2] Fogarty International Center, National Institutes of Health,

16 Center Drive, Bethesda, MD, 20892, USA

[3] National Institute of Public Health,

Center for Evaluation Research and Surveys, Cuernavaca, Mexico

[4] University of California, Berkeley, USA

1 Derivation of the basic reproduction number

From our age-structured model given in the main text, the expression for R_0 can be derived using the next-generation operator method (1; 2). That is, $R_0 = r(FW^{-1})$, i.e., R_0 is the spectral radius of the next generation matrix FW^{-1}. In our model,

$$F = \begin{pmatrix} 0_{6\times 6} & B & B \\ 0_{12\times 6} & 0_{12\times 6} & 0_{12\times 6} \end{pmatrix},$$

with

$$B(i,j) = \beta_{ij}\xi_i \;\; \text{where } \xi_i = \frac{N_i}{N} \;\; \text{for } i,j = 1,\cdots,6,$$

and

$$W = \begin{pmatrix} kI_{6\times 6} & 0_{6\times 6} & 0_{6\times 6} \\ -kI_{6\times 6} & W_1 & 0_{6\times 6} \\ 0_{6\times 6} & W_2 & W_3 \end{pmatrix},$$

where for $i, j = 1, \cdots, 6, \quad j \neq i$,

$$W_1(i,i) = \alpha_i + \gamma_1, \quad W_1(i,j) = 0,$$

$$W_2(i,i) = -\alpha_i, \quad W_2(i,j) = 0,$$

$$W_3(i,i) = \gamma_2 + \delta_i, \quad W_3(i,j) = 0.$$

Therefore the next generation matrix is given by

$$FW^{-1} = \begin{pmatrix} M_{ij} \end{pmatrix}_{i,j=1,\cdots,6}$$

with

$$M_{ij} = \beta_{ij}\xi_i[(\frac{1}{\alpha_j + \gamma_1})(1 + \frac{\alpha_j}{\gamma_2 + \delta_j})], \quad i, j = 1, \cdots, 6.$$

It is important to note that R_0 depends on the age distribution of the initial susceptible population $\xi_k = \frac{N_k}{N}$ where $\sum_{k=1}^{6} \xi_k = 1$.

References

[1] Diekmann O, Heesterbeek J. Mathematical Epidemiology of Infectious Diseases: Model Building, Analysis and Interpretation. Wiley, New York; 2000.

[2] van den Driessche P, Watmough J. Reproduction numbers and sub-threshold endemic equilibria for compartmental models of disease transmission. Math. Biosci. 2002;180:29-48.
